# Tissue engineering approaches for lymphedema: biomaterial innovations and clinical potential

**DOI:** 10.3389/fcell.2025.1537050

**Published:** 2025-04-15

**Authors:** Pooja Deshpande, Maya Dornbrand-Lo, Varoon Phondge, Patrick Kelly, Alex K. Wong

**Affiliations:** Rutgers New Jersey Medical School, Division of Plastic and Reconstructive Surgery, Newark, NJ, United States

**Keywords:** lymphedema, lymphatics, tissue engineering, bioengineering, biomaterials

## Abstract

The lymphatic system plays a critical role in maintaining fluid balance and immune regulation. Lymphedema, and other lymphatic disorders, highlight the need for advanced therapeutic approaches, including tissue engineering. This review examines the latest developments in artificial lymphatic tissue engineering, focusing on scaffold materials, lymphangiogenic factors, and regenerative strategies to replicate the intricacy of lymphatic vessels and nodes. We conducted a thorough literature review of current practices and applications in lymphatic tissue engineering. Findings show that biomaterials such as hydrogels, decellularized matrices, and synthetic polymers provide effective scaffolds for lymphatic endothelial cell proliferation and lymphangiogenesis. Advances in growth factor delivery and stem-cell based therapies have further enhanced the viability of engineered lymphatic tissues. Despite promising progress, challenges in achieving functional replication of lymphatic structures and clinical translation of research remain. Ongoing research must address scaffold biocompatibility, optimized growth factor targeting, and scalable production to advance therapeutic options for lymphatic disorders. This review underscores the potential for transformative patient outcomes through innovative bioengineering solutions.

## 1 Introduction

### 1.1 The lymphatic system

The lymphatic system is an integral part of the human body and is involved in various physiologic processes, including circulatory, metabolic, and immune functions. The many functions of the lymphatic system allow the body to maintain homeostasis. One primary function of the lymphatic system is to collect and return protein-rich, solute-rich interstitial fluid back to blood circulation. Another important function of the lymphatic system is to aid in a physiologic immune response by transporting foreign antigens and immune cells to the lymph nodes (Sung et al., 2022). The lymphatic system consists of several key components. The lymphatic capillaries are small, highly permeable vessels that are structured differently from circulatory vasculature. Unlike vascular endothelial cells, the lymphatic endothelial cells (LECs) line the lymphatic capillaries in a single layer with discontinuous, button-like junctions. These gaps in the basement membrane contribute to the highly permeable nature of the lymphatic capillaries and permit the uptake of “lymph” or interstitial fluid (Jia et al., 2021). Specialized anchoring filaments attach the LECs to the surrounding extracellular matrix, while also keeping the capillaries open to allow for permeability (Butler et al., 2009). The lymphatic capillaries merge to form larger collecting lymphatic vessels, characterized by a continuous basement membrane with smooth muscle cells and semilunar-shaped valves. Together, these structures allow for the unidirectional flow of lymphatic fluid (Sung et al., 2022). The collecting vessels transport lymphatic fluid to the lymph nodes, an encapsulated organ that serves to filter lymph and house immune cells. Lymph nodes are categorized into two groups based on their location within the body: peripheral lymph nodes, which collect lymph from all regions of the body, and mesenteric lymph nodes, which are involved in the immune response of the intestines (Nosenko et al., 2016). The collecting lymphatic vessels also converge to form larger lymphatic trunks and ducts, of which the main thoracic duct returns the lymphatic fluid back into venous circulation via an anastomosis through the cardinal vein (Butler et al., 2009). The thoracic duct, the largest lymphatic vessel in the body, originates from the cisterna chyli in the abdomen and ascends through the thorax before draining into the venous system at the junction of the left subclavian and internal jugular veins (Ilahi et al., 2025). This return of lymphatic fluid is critical for maintaining fluid homeostasis and immune function, as disruptions in this process can lead to conditions such as lymphedema.

### 1.2 Lymphedema

**FIGURE 1:**
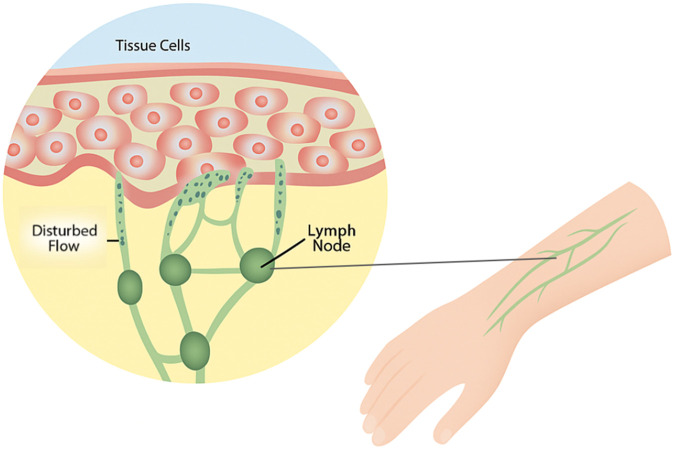
Visualization of disrupted lymphatic flow in lymphedema. Figure illustrates the disruption of fluid flow within tissue and lymphatic vessels. This disruption causes fluid to accumulate in tissue spaces, leading to swelling, particularly in the limbs. Over time, chronic fluid buildup promotes fibroadipose tissue deposition, triggering an inflammatory response that further obstructs lymphatic drainage.

The unique and delicate architecture of the lymphatic system makes it particularly prone to disease when its structure is compromised. Lymphedema is a chronic, progressive condition, characterized by the accumulation of lymphatic fluid in the extracellular space due to dysfunction of the lymphatic system. This build-up of fluid leads to tissue swelling, often in the limbs, and chronic deposition of fibroadipose tissue (Brown et al., 2023), which hardens affected tissue, and further impedes lymphatic drainage. This fluid stasis also triggers an inflammatory response. Figure 1 illustrates the disrupted lymphatic flow and tissue swelling seen in lymphedema patients. The innate and adaptive immune responses play critical roles in the pathophysiology of lymphedema. Innate immune mechanisms are activated following lymphatic injury, leading to chronic inflammation. Danger-associated molecular patterns (DAMPs) are released from damaged lymphatic endothelial cells, activating Toll-like receptors (TLRs) and initiating pro-inflammatory cascades. Macrophages are then heavily recruited to the affected tissue, where they produce inflammatory cytokines such as IL-6 and TGF-β, contributing to fibrosis and tissue remodeling (Kataru et al., 2019). While macrophages initially promote lymphangiogenesis through VEGF-C secretion, they also contribute to impaired lymphatic function by releasing nitric oxide, reducing lymphatic vessel contraction and increasing fluid stasis (Yuan et al., 2019). The adaptive immune response is also dysregulated in lymphedema, with a predominant CD4^+^ T cell-mediated inflammatory reaction. Persistent T helper 2 (Th2) response promotes fibrosis by increasing expression of profibrotic growth factors such as IL4, IL-13, and TGF-β1, along with reducing lymphangiogenesis, and impairing lymphatic transport (Kataru et al., 2019). Regulatory T cells (Tregs) also accumulate in lymphedematous tissue, additionally suppressing innate and adaptive immune responses and contributing to local immunosuppression, thus increasing susceptibility to infections (García Nores et al., 2018).

Lymphedema can have different etiologies. It is broadly classified into primary and secondary forms. Primary lymphedema is a rare disease, occurring in about 1 in 100,000 individuals (Sleigh et al., 2024). It is characterized by a genetic abnormality that impairs the development of the lymphatic system and weakens its drainage capacity. In contrast, secondary lymphedema is more common and typically results from external damage to the lymphatic system (Drobot et al., 2024). It affects approximately 1 in 1,000 Americans (Sleigh et al., 2024) and about 250 million people worldwide (Schulze et al., 2018). Globally, secondary lymphedema is most commonly caused by infections such as filariasis, while in Western countries, it is frequently attributed to cancer treatments, including lymph node dissection and radiation therapy. This condition is particularly common among breast cancer patients who have undergone mastectomy (Drobot et al., 2024). Regardless of its cause, lymphedema leads to progressive fluid accumulation, which can result in discomfort, restricted movement, loss of function, increased risk of infection, and a significant impact on a patient’s quality of life (Brown et al., 2022).

### 1.3 Therapies for lymphedema

**FIGURE 2:**
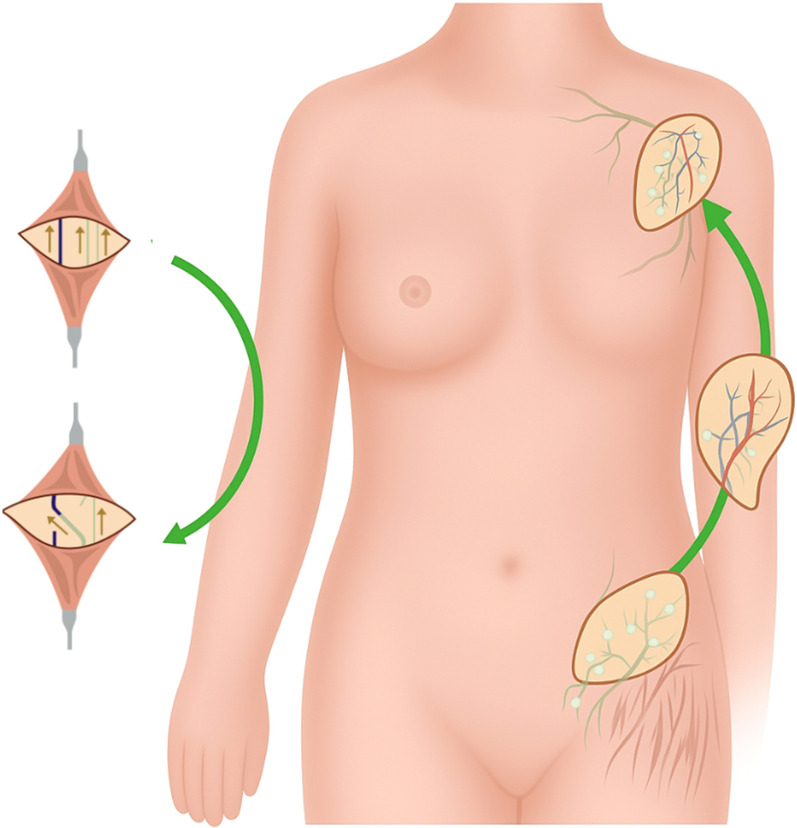
Current surgical therapies for lymphedema. Figure illustrates current microsurgical treatments for lymphedema, including lymphaticovenular anastomosis (LVA) and vascularized lymph node transfer (VLNT). LVA bypasses obstructed lymphatic pathways by connecting lymphatic vessels to nearby veins, facilitating fluid drainage into the venous system. VLNT involves transplanting functional lymph nodes from a donor site to a recipient site to restore lymphatic drainage and reduce swelling.

Despite the fact that secondary lymphedema is common and morbid, there is no effective treatment. Current therapies often focus on minimizing symptoms such as pain and swelling yet do not offer a definitive cure. The gold standard therapy for lymphedema is complete decongestive therapy: a combination of manual lymphatic drainage, daily bandaging, skin care, exercising, and compression garments (Shaitelman et al., 2015). However, the effect of this treatment has been doubted in recent years (Drobot et al., 2024). In severe or refractory cases, further pharmacologic or surgical intervention is needed. In experimental models, pharmacologic therapies focus on stimulating lymphangiogenic factors, such as vascular endothelial growth factor-C (VEGF-C) and fibroblast growth factor 2 (FGF2). Other therapies include anti-inflammatory agents, such as tacrolimus, which works to inhibit CD4^+^ cell proliferation, and anti-fibrotic agents, such as pirfenidone, which works against transforming growth factor beta-1 (TGF-β1), a key regulatory of fibrosis (Brown et al., 2023). It should be noted, that to date, there is no approved pharmacologic therapy for lymphedema. Surgical intervention aims to restore normal lymphatic function through the development of alternative drainage methods such as lymphaticovenular anastomosis (LVA) or vascularized autologous lymph node transfer (VLNT), which have been depicted in Figure 2, however these procedures do not correct the fibroadipose accumulation (Drobot et al., 2024). The addition of liposuction to these procedures has shown to help with the removal of fibroadipose tissue (Brazio and Nguyen, 2021). While surgical intervention can provide symptomatic relief, procedures are often lengthy and complex, with an additional risk of inadvertently inducing lymphedema at the donor lymph node harvest site (Schaverien et al., 2018). The emerging field of tissue engineering holds potential for treating lymphedema by using lymphatic grafting to reconstruct damaged vasculature (Kanapathy et al., 2014). This new strategy aims to promote lymphangiogenesis and create functional lymphatic tissue *in vitro* for transplantation (Jia et al., 2021).

This review centers on identifying the processes involved in the development of artificial lymphatic tissue. By examining the architecture and physiological functions of natural lymphatic tissue, we aim to deepen our understanding of the foundational principles. Additionally, we assess the current biomaterials available for tissue generation and explore the challenges, recent advancements, and future implications of this emerging technology.

## 2 Development and structure of the lymphatic system

### 2.1 Embryonic development

Two theories have dominated much of the discourse regarding the embryonic development of the lymphatic system. One theory suggests that LECs originate from veins and spread outwards to form the lymphatic system (Sabin, 1902). Another theory suggests that LECs develop from mesenchymal cells in tissue and eventually connect to the venous system (Null et al., 2024). However, recent studies have indicated that lymphatic vessel formation is complex, and can have both venous and non-venous origin, depending on the organ and the species (Semo et al., 2016). During embryonic development, lymphatic vessels develop after circulatory vasculature, at approximately 6 weeks after fertilization. The precursory endothelial cells that line this system are derived from the embryonic cardinal veins. Lymphatic vessels form in a similar fashion to blood vessels–creating lymphovenous and intra-lymphatic anastomoses. Approximately 8 weeks post-fertilization, six primary lymph sacs form: the cisterna chyli, located in the abdomen; the retroperitoneal lymph sac, located behind the abdomen; two iliac lymph sacs, located in the pelvic region; and two jugular lymph sacs, located in the neck region. Lymphatic vessels then form from these lymph sacs in a process of self-proliferation and sprouting to help connect the sacs to one another and to other parts of the body. Migrating mesenchymal cells enter these lymph sacs and contribute to the formation of lymphatic networks, connective tissue, and other layers of the lymph sacs. During the fetal period, these lymph sacs develop into clusters of lymph nodes (Null et al., 2024). Nearby proximal nerve fibers release retinoic acid (RA) to signal the mesenchymal cells of the lymph sacs to produce CXCL13 chemokine. CXCL13 plays a crucial role in lymph node development by attracting lymphoid tissue inducer cells (LTiCs), which are essential for the formation of the lymph node. In the absence of CXCL13 or its receptor CXCR5, peripheral lymph nodes fail to develop, (van de Pavert et al., 2009). The LTiCs also express lymphotoxin-β to interact with lymphoid tissue organizer (LTo) cells, aiding in lymph node development (Hampton and Chtanova, 2019). As the lymph node matures, distinct T and B cell zones emerge. T cell development begins with hemopoietic stem cells (HSCs) in the fetal liver and bone marrow. A subset of HSCs produces recombination activating gene 1 and 2 (RAG1 and RAG2) to eventually become common lymphoid progenitor (CLP) cells. A subset of these cells then migrates to the thymus to differentiate into early thymic progenitors (ETP) cells, which can be used to generate B cells, T cells, Natural Killer cells, myeloid cells, and dendritic cells. Specifically, B and T cells are attracted and matured through the expression of chemokines CCL19, CCL21, and CXCL13 produced by follicular dendritic cells (FDCs). These chemokines facilitate the movement of B cells into the marginal zone and T cells into the cortex for the formation of the respective B and T zones (Cano et al., 2013).

### 2.2 Lymph node anatomy

**FIGURE 3:**
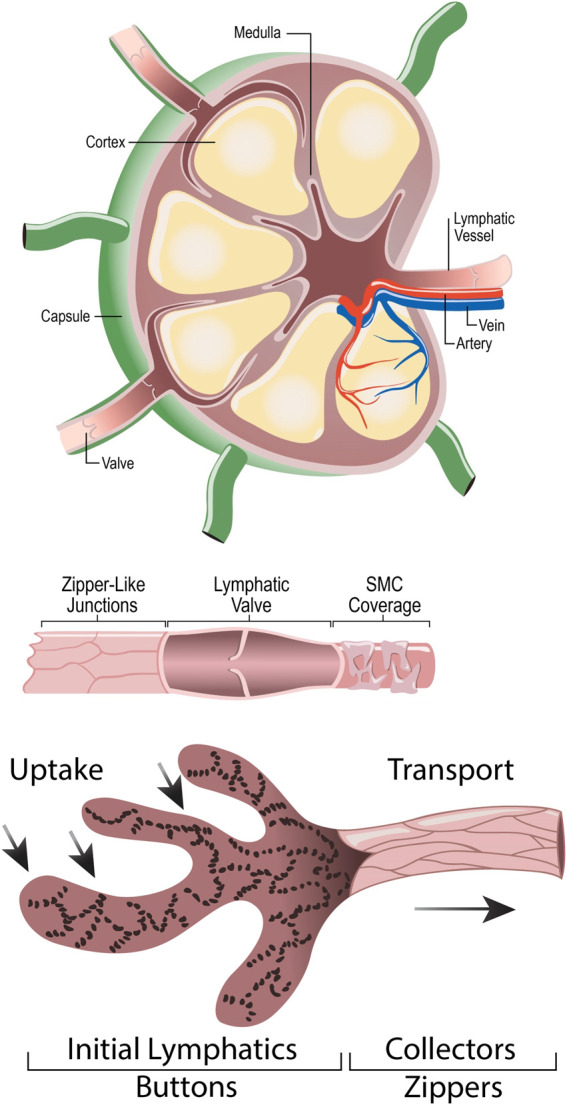
Anatomy of Lymph Node **(A)**, Lymphatic Vessel **(B)**, and Lymphatic button-like junctions **(C)**. **(A)** depicts the anatomy of a lymph node, highlighting the lymphatic vessels and valves, artery, vein, capsule, cortex, and medulla. **(B)** depicts a lymphatic vessel, highlighting the unidirectional lymphatic valve, zipper-like junctions, and smooth muscle cell coverage. **(C)** amplifies the button-like junctions found between lymphatic endothelial cells.

Lymph nodes are small, encapsulated organs, found all throughout the body. The structure of a lymph node facilitates its functions as a filter and a site for immune responses. Lymph nodes are found along lymphatic vessels, receive fluid from an afferent lymphatic vessel, and expel fluid out through an efferent lymphatic vessel (Null et al., 2024). Lymph nodes are composed of layers, including the capsule, the subcapsular sinus, the cortex, the paracortex, and the medulla. The capsule is composed of dense connective tissue and collagenous fibers. The subcapsular sinus allows for the transportation of lymphatic fluid from the capsule to the cortex. The cortex houses B-cell lymphoid follicles. When presented with an antigen, immature B-cells form a germinal center. Dendritic cells and resting B-cells together surround the germinal center to create a mantle zone. Between the B-cell lymphoid follicles lies the paracortex, which serves as a site for lymphocyte trafficking and houses T-lymphocytes. Dendritic cells also interact with T-cells in this layer by presenting antigens. The innermost portion of the lymph node is the medulla, which contains blood vessels, medullary sinuses, and medullary cords. Medullary sinuses act as a channel for lymphatic flow and drain fluid out of an efferent vessel, while medullary cords house macrophages and plasma cells that contribute to later stages of the immune response (Jia et al., 2021; Bujoreanu and Gupta, 2024). A conduit system in the lymph node helps to quickly deliver soluble antigens, immune complexes, and dendritic cells from the subcapsular sinus to phagocytic cells and post-capillary venules (Witte et al., 2006). Figure 3 visualizes the anatomy of the lymph node, lymphatic vessel, and lymphatic junctions.

### 2.3 Molecular regulation and lymphangiogenesis

Various signaling pathways and molecules are necessary in guiding proper lymphatic vessel formation, or lymphangiogenesis. While relevant for embryonic development, this process can also be pathologic in causing tumor metastasis and cancer growth. In the early stages of development, many factors play a role in ensuring lymphatic cell specification. Before differentiating into lymphatic-specific cells, markers such as lymphatic vessel endothelial hyaluronan receptor 1 (Lyve1) are induced in a subpopulation of venous endothelial cells to signify lymphatic fate (Butler et al., 2009). Yet, while this marker has early expression in lymphatic progenitor cells, studies have shown that the absence of Lyve1 in mice results in an apparently normal phenotype, with no obvious differences in lymphatic vessel structure or function. This finding suggests that while Lyve1 is an early marker for LECs, it is not essential for baseline lymphatic function, possibly due to the compensatory mechanism of various other factors involved in lymphangiogenesis (Gale et al., 2007). Prox1 is a key transcription factor that directs lymphatic cell fate (Semo et al., 2016). Studies have shown that Prox1 knock-out (KO) mice lack lymph sacs and lymphatic vessels (Wigle and Oliver, 1999). Prox1 regulation, and therefore LEC specification, is also affected by other transcription factors, including Sox18 and Coup-TFII, while secreted factors such as Wnt5b induce and Bmp2b inhibit the process (Semo et al., 2016). After specification, lymphangiogenic growth factors such as VEGF-C and VEGF-D induce lymphatic vessel proliferation. While both factors bind to the specific lymphatic endothelial cell receptor VEGFR-3, VEGF-C binds at a higher affinity (Witte et al., 2006). Studies showed that mice who lacked VEGFR-3 died early *in utero*, prior to lymphatic development, while mice who lacked VEGF-C did not develop lymph sacs (Karkkainen et al., 2004). Activation of VEGFR-3 leads to phosphorylation of serine kinases Jun N-terminal kinase (JNK), AKT, and extracellular signal-regulated kinase (ERK) to promote the proliferation, migration, and survival of LECs (Jia et al., 2021; Coso et al., 2012). FGF1 and FGF2 come from a family of fibroblast growth factors and are involved in angiogenesis and lymphangiogenesis. FGF2 has been shown to bind at low- and high-affinity receptors on LECs to promote the proliferation, migration, and survival of these LECs. An important receptor from this family, FGFR-3, also serves as a target gene for Prox1, and therefore, lymphatic development (Shin et al., 2006). Another family of growth factors, known as the platelet-derived growth factors (PDGF) are involved in this process. PDGF-BB is a growth factor that has directly been linked to lymphangiogenesis. Its receptors PDGFRα and PDGFRβ are expressed in LECs and are capable of inducing lymphangiogenesis as well (Jitariu et al., 2015). Though many of these factors work together to promote lymphangiogenesis, some have shown undesirable effects, specifically in the setting of cancer cell proliferation. Understanding the complex relationship between compounds and molecules in lymphangiogenesis can help promote vessel formation in engineered tissue.

## 3 Biomaterials and engineering

### 3.1 Scaffolds

A stable, structural scaffold creates the foundation for an environment conducive for cell growth, differentiation, and spatial organization. In normal embryonic development, stromal cells create this foundation out of polymeric collagen fibers. In artificial tissue engineering, various biomaterials have been identified to create a structural framework on which cells can grow (Nosenko et al., 2016). The primary function of this scaffold is to provide a surface for cell proliferation while delivering essential nutrients to the cells. In some instances, scaffolds have been shown to maintain their structure for up to 1 year after transplantation. When selecting a scaffold, it is important that the material is biocompatible, and therefore is non-toxic, and does not cause an immune response in the host body. Ideally, a scaffold should also be biodegradable at a rate that matches tissue formation, in order to leave behind functional tissue. Scaffolding should have the strength and flexibility to withstand physical and mechanical forces (Sung et al., 2022; Kanapathy et al., 2014; Alderfer et al., 2018). These materials can be broadly categorized into two groups: natural materials and synthetic materials (Kwon et al., 2018). In this review, we will highlight the many different biomaterials that have been used as scaffolding in artificial tissue engineering, which have been summarized in Table 1.

**TABLE 1:** Summarizes the various biomaterials used for lymphatic tissue engineering. The materials are categorized broadly into hydrogels, decellularized extracellular matrices, biodegradable polymers, and non-biodegradable polymers. Various examples of these materials used in literature have been summarized in the table, along with the corresponding references.

Technique	Methods	Model system	Results	Ref.
Hydrogels				
Fibrin	Human dermal BECs and LECs were combined with recombinant VEGF, varying concentrations of fibrin and collagen, and slow interstitial flow for 10 days	*In vitro*	LECs favored fibrin-only matrix, while BECs favored collagen-containing matrix; BECs organized into thick, branching, wide networks; LECs organized into slender, fine networks	Helm et al. (2007)
Collagen				
Nanofibrillar Collagen Scaffold (NCS)	Systematic review of studies using biomaterials for treatment of primary and secondary lymphedema	N/A	NCS showed average excess limb volume reduction of 1%–10.7%, and lymphangiogenesis on imaging	Drobot et al. (2024)
	Evaluating change in limb volume and lymphangiogenesis in prophylactic NCS group, delayed NCS treatment group, and control group in a rat hindlimb lymphedema mode	*In vivo*	Prophylactic group showed no increase in affected limb volume size while the delayed treatment group showed a reduction in affected limb volume	Nguyen et al. (2022)
Gelatin	Used gelatin-based hydrogels containing VEGF-C for cardiac remodeling in myocardial ischemia mouse model	*In vivo*	VEGF-C augments endogenous lymphangiogenesis and cardiac remodeling	Shimizu et al. (2018)
Hyaluronic Acid (HA)	Human LECs were seeded onto varying stiffness HA-hydrogels to evaluate the influences on LEC behavior and tube formation	*In vitro*	Low matrix elasticity paired with high concentrations of VEGF-C supports lymphatic cord-like structure formation; decreased matrix stiffness upregulates key lymphatic markers	Alderfer et al. (2018)
	VEGF-C and ANG-2 were loaded onto HA and methylcellulose hydrogels to evaluate lymphangiogeneic signaling *in vitro*, and vascular permeability in a sheep lymphedema model	*In vitro* and *in vivo*	Lymphangiogenic signaling was induced in target endothelial cells; treatment group had increased lymphatic function and reduced edema	Baker et al. (2010)
Matrigel	Matrigel scaffold was used to deliver hMAPCs to a transplanted lymph node in a mouse model	*In vivo*	hMAPCs mixed with Matrigel enhanced survival of transplanted lymph nodes, promoted lymphangiogenesis, and improved lymphatic drainage	Beerens et al. (2018)
Decellularized Extracellular Matrix (dECM)				
Decellularized lymph nodes (dLNs)	Lymph nodes from adult mice were harvested to identify an optimal method of decellularization, and then were replanted into renal capsule of mice to test antigenicity; also repopulated by splenocytes to test cellular delivery	*In vitro* and *in vivo*	Implanted decellularized lymph nodes did not elicit a significant immune response; repopulated lymph nodes resulted in successful *in vivo* cellular delivery	Cuzzone et al. (2015)
	dLNs were recellularized with BMDCs isolated from mouse femurs and tibias and reimplanted to induce antitumor immunity	*In vitro* and *in vivo*	BMDC-loaded lymph nodes successfully rejected, E.G.,7-OVA tumors in mice	Lin et al. (2019)
	dLNs from rats were recellularized with hADSCs to study the effects on lymphangiogenesis in a rat lymphedema model	*In vitro* and *in vivo*	VEGF-A and LYVE-1 were highly expressed in recellularized lymph nodes; recellularized lymph nodes were more successful in inducing lymphangiogenesis compared to stem cells alone or dLNs alone	Kang et al. (2023)
Decellularized arterial scaffold	hADSCs were differentiated into lymphatic-like endothelial cells and seeded onto decellularized arterial scaffold to construct lymphatic vasculature	*In vitro* and *in vivo*	Isolated hADSCs seeded onto decellularized arterial scaffolding grew successfully	Yang et al. (2018)
Bioedegradable Polymer				
Polyglycolic Acid (PGA)	LECs combined with PGA scaffold were implanted into mice	*In vitro* and *in vivo*	LECs can be used as seed cells, and LEC-PGA compounds shows preliminary characteristics of lymphatic vessels	Dai et al. (2010)
	Various 3D PGA scaffolding structures were created to determine most effective support system	*In vitro*	Plain 3D PGA scaffolds are most effective for supporting tissue regeneration	Kundak and Bilisik (2023)
Non-biodegradable Polymer				
Polyhedral oligomeric silsesquioxane poly (carbonate-urea) urethane (POSS-PCU)	Review on lymphatic tissue engineering	N/A	POSS-PCU has been successful in cardiovascular applications and shows promise in lymphatic tissue engineering because of its biocompatibility, durability, and biofunctionality	Kanapathy et al. (2014)
Silicone Tubes	Systematic review of studies using biomaterials for treatment of primary and secondary lymphedema	N/A	Silicone tube implantation showed reduction of limb volume and circumference in patients with advanced lymphedema	Drobot et al. (2024)

### 3.2 Natural materials

#### 3.2.1 Hydrogels

**FIGURE 4:**
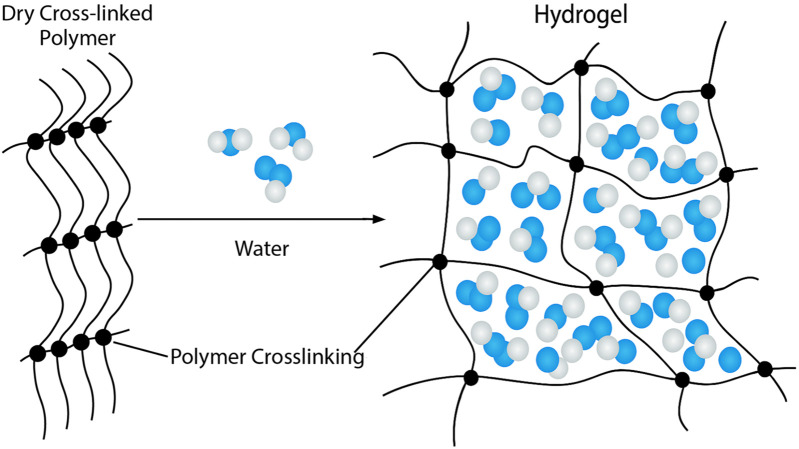
Structure of Hydrogels. Hydrogels consist of a three-dimensional, crosslinked polymer network. This structure is favorable for absorbing large amounts of water, resulting in a highly porous, soft, and flexible structure that closely mimics the extracellular matrix.

Hydrogels are three-dimensional, crosslinked polymer structures that absorb large amounts of fluid and exhibit high biocompatibility, shown in Figure 4. Their high-water content, high porosity, flexibility, and soft structure closely resemble the extracellular matrix (ECM) of living tissue and are often selected for lymphatic tissue engineering (Kwon et al., 2018; Ho et al., 2022). Hydrogels were first developed as a biomaterial in 1960 by Wichterle and Lim and were initially used as a filler for eye enucleation and contact lens. Since then, hydrogels have been used in a variety of fields, including agriculture, drug delivery, food industry, and tissue engineering (Ho et al., 2022). Hydrogels have been shown to generate functional lymphatic vessels (Alderfer et al., 2018). Many natural polymers, including fibrin, collagen, gelatin, hyaluronic acid, and matrigel, have been used to construct hydrogel scaffolds.

Fibrin is a natural polymer commonly used in hydrogels. When engineered into a fibrin-VEGF fusion protein, interstitial fluid flow significantly enhanced capillary formation in both lymphatic (LECs) and blood endothelial cells (BECs). However, while fibrin supports LEC growth, it is less stable as a matrix compared to collagen. Collagen, a fibrous protein that is a major component of the extracellular matrix (ECM), provides more stability for long-term use. One study created matrices with varying combinations of fibrin and collagen, exposed to both LECs, BECs, and VEGF, and showed that both polymers promote vasculogenesis, with LECs demonstrating a preference for the fibrin-only matrix (Sung et al., 2022; Helm et al., 2007).

Nanofibrillar collagen scaffolds (NCSs) are a specialized form of collagen scaffolding that have demonstrated promising potential in the treatment of lymphedema. These implantable, biocompatible mesh ribbons are made from purified type 1 porcine collagen. Serving as soft reinforcements, they can be used to connect healthy tissues while promoting the release of local growth factors, which in turn supports lymphatic regeneration. In clinical application, NCS has been shown to reduce average excess limb volume by up to 10.7%, with additional evidence of lymphangiogenesis on imaging (Drobot et al., 2024). In a rat lymphedema model, treatment with NCS at the time of lymph node removal prevented the development of lymphedema. This was demonstrated by the absence of limb volume increase in the prevention group, as confirmed by computed tomography-based volumetric analysis. Additionally, near-infrared (NIR) fluoroscopy showed the formation of new functional lymphatic vessels, suggesting that NCS facilitated lymphatic regeneration and guided lymph flow towards healthy lymphatics. Treatment with NCS and ADSCs in established lymphedema reduced the burden of lymphedema in the rat model (Nguyen et al., 2022).

Gelatin is a denatured form of collagen that is biocompatible and easily accessible. In one study, gelatin-based hydrogels containing VEGF-C were used to augment lymphangiogenesis in mice subjected to myocardial ischemia. This treatment was shown to reduce inflammation and edema while improving cardiac function, suggesting that lymphangiogenesis can offer a therapeutic role in cardiovascular disease (Shimizu et al., 2018).

Hyaluronic acid (HA) is a polysaccharide that is another naturally occurring component of the ECM. In addition to being involved with tissue engineering, HA also plays a critical role in wound healing. HA interacts with the receptor LYVE-1 found in LECs to induce cell migration and lymphangiogenesis as it breaks down. LECs cultured in HA hydrogels also demonstrated the ability to form lymphatic cord-like structures (Alderfer et al., 2021). In a sheep model, a mixture of HA and methylcellulose gel-based drug delivery system released lymphangiogenic growth factors VEGF-C and angiopoietin-2 (ANG-2), which helped treat lymphedema (Sung et al., 2022; Baker et al., 2010).

Matrigel, derived from mouse tumors, is a mixture of ECM proteins such as laminin, collagen IV, and enactin. In lymphatic research, Matrigel has supported lymphatic capillary growth and network regeneration when combined with stem cells in mouse models (Sung et al., 2022). One study showed that a Matrigel scaffold was used to deliver human multipotent adult progenitor cells (hMAPCs) in a lymph node transplantation mouse model. Matrigel was shown to enhance the survival of transplanted lymph nodes and promote the formation of lymphatic vessels. The hMAPCs that were introduced in the model helped connect transplanted lymph nodes to the host lymphatic network, which therefore significantly improved lymphatic drainage (Beerens et al., 2018).

#### 3.2.2 Decellularized extracellular matrix

Decellularized extracellular matrices (dECM) have a prominent hold in tissue engineering, as they have been used to regenerate various organs, including the liver (Neishabouri et al., 2022; Dai et al., 2022), lung (Gilpin et al., 2014), and heart (Sánchez et al., 2015). The advantage of dECMs is their accessibility, natural biochemical environment, and lack of immunogenicity in host tissue. The decellularization process produces a natural ECM, which houses biological factors, like growth factors and cytokines, and an architectural structure resembling the original tissue, that can eventually be repopulated with host cells (Yang et al., 2022; Lin et al., 2019; Mazza et al., 2015).

Several studies have demonstrated the effectiveness of decellularized extracellular matrix (dECM) in generating artificial lymph nodes and lymphatic vessels. In 2015, Cuzzone et al. successfully decellularized murine lymph nodes, creating scaffolds that were repopulated by host splenocytes. These scaffolds maintained the native extracellular matrix architecture, showed no immunogenicity, and had the potential to deliver leukocytes *in vivo* (Cuzzone et al., 2015). Following this, another study highlighted the ability of decellularized lymph nodes to serve as a supportive scaffold for bone marrow dendritic cells (BMDCs). When transplanted into mouse models, these BMDC-loaded scaffolds successfully triggered an anti-tumor response, leading to the rejection of, E.G.,7-OVA tumors (Lin et al., 2019). More recently, human adipose-derived stem cells (hADSCs) were injected into decellularized lymph nodes, producing recellularized scaffolds that were then transplanted into a mouse lymphedema model. This study found that the recellularized scaffold stimulated greater lymphangiogenesis compared to either stem cells alone or the decellularized matrix alone. This suggests a promising new treatment for lymphedema, as it promotes the formation of new lymphatic vessels and restores lymphatic function. By addressing the root causes of lymphedema rather than merely managing its symptoms, this approach offers the potential for more effective and long-lasting treatment (Kang et al., 2023). Yang et al. demonstrated that human adipose-derived stem cells (hADSCs) can also generate lymphatic endothelial-like cells. When combined with a decellularized arterial scaffold, these cells successfully engineered a lymphatic vessel, which could potentially be used to create a lymphatic graft (Yang et al., 2018).

### 3.3 Synthetic materials

#### 3.3.1 Biodegradable polymers

Synthetic biodegradable polymers, such as polyglycolic acid (PGA) and poly (lactic-co-glycolic acid) (PLGA), can also be used as scaffolds. PGA has promising applications in lymphatic tissue engineering (Sung et al., 2022), while PLGA has proven useful in skin tissue engineering (Kumbar et al., 2008). These scaffolds are highly customizable, allowing researchers to replicate specific cellular environments and adjust features such as degradation rates for targeted applications (Sung et al., 2022; Kanapathy et al., 2014). Dai et al. demonstrated that a PGA scaffold seeded with lymphatic endothelial cells (LECs) facilitated the preliminary formation of lymphatic vessels (Dai et al., 2010). Recent studies have also explored different PGA scaffold structures, including 3D plain, semi-interlaced, and orthogonal woven designs, concluding that 3D plain PGA fibers are most effective as a temporary supportive structure for tissue regeneration (Kundak and Bilisik, 2023).

#### 3.3.2 Non-biodegradable polymers

Unlike biodegradable polymers, which are temporary, non-biodegradable polymers are strong and maintain structural integrity. One such polymer, polyhedral oligomeric silsesquioxane poly (carbonate-urea) urethane (POSS-PCU), shows promise in lymphatic graft engineering. It has already been successfully incorporated in heart valves and bypass grafts due to its biocompatibility, durability, antimicrobial properties, and ability to support endothelialization, making it a potential candidate for engineering lymphatic grafts (Kanapathy et al., 2014). Silicone tubes, though not used for bioengineering purposes, are non-biodegradable materials that can be used to generate an artificial track across regions of scarred or irradiated tissue. As a result, silicone tubes have been used in lymphedema patients as a less invasive option to help reduce limb volume (Drobot et al., 2024).

### 3.4 Stem cells for lymphatic tissue engineering

Stem cells play a crucial role in regenerative medicine, which aims to repair or replace damaged tissues and organs. Understanding the different types of stem cells and their role in tissue regeneration is essential for bioengineering lymphatic tissue. Stem cells are undifferentiated cells that possess the potential to specialize into various cell types and self-renew. While stem cell therapy alone faces considerable limitations due to poor cell survival after transplantation, tissue engineering has overcome these challenges by creating supportive environments that encourage stem cell viability, growth, and differentiation (Kwon et al., 2018). The most fundamental stem cell is the embryonic stem cell (ESC). ESCs are pluripotent cells derived from blastocysts and are capable of differentiating into any cell type in the body (Sung et al., 2022). These cells hold immense potential for tissue regeneration due to their pluripotency but are faced with many obstacles. Ethical issues have delayed the use of ESCs in clinical practice because they originate from human embryos. It is also difficult to induce 100% of ESCs into differentiated cells, and thus the cells that remain undifferentiated face the risk of forming a teratoma (Kwon et al., 2018). Additionally, these cells are allogenic and could potentially subject the patient to an immune reaction following transplantation (Boyd et al., 2012). Induced pluripotent stem cells (iPSCs) address the ethical concerns of ESCs, as they are adult somatic cells that are reprogrammed to a pluripotency similar to ESCs. Another advantage of using iPSCs in research is that they are patient-derived, reducing potential immune responses; however, the potential of generating tumors still remains a risk (Li et al., 2017). Hematopoietic stem cells (HSCs) are oligopotent, meaning they can differentiate into a few specific cell types, in this case, blood and immune system cells. In mice, these cells have been shown to give rise to LECs that eventually integrate into lymphatic vessels of tissues such as the kidney, liver, stomach, and intestines. Given their ability to form LECs and lymphatic vessels, HSCs can serve as a valuable tool for regenerating damaged or deficient lymphatic tissue. In addition to aiding in normal lymphatic endothelium, it has also been shown that HSCs can be incorporated into tumor-associated lymphatic vessels. Therefore, HSCs could also serve as a potential target for attenuating lymphatic system mediated tumor metastasis (Jiang et al., 2008). Multipotent adult progenitor cells (MAPCs) have the ability to differentiate into cell types of a specific organ, and when cultured with VEGF-A, they even separate into a mixture of arterial, venous, and lymphatic endothelial cells (Sung et al., 2022). Adipose-derived stem cells (ADSCs) are a group of mesenchymal stem cells that are specifically found in adipose tissue and are a promising source of lymphangiogenesis. Two mechanisms have been proposed for promoting lymphangiogenesis: paracrine secretion of growth factors and direct differentiation into LECs (Forte et al., 2021). While ADSCs have a strong potential for use in lymphatic tissue engineering, further studies are still needed to understand the long-term effects of their uses (Sung et al., 2022). While challenges remain for the use of stem cells in tissue engineering, the field is rapidly evolving. With an understanding of the fundamental cells needed in bioengineering artificial tissue, advancements can be made in biomaterials and the construction of lymphatic vessels to help revolutionize treatment for lymphedema and other lymphatic diseases.

### 3.5 3D bioprinting for lymphatic tissue engineering

3D bioprinting offers a promising approach to create multi-layered structures that mimic the complexity of natural tissues. This technology has been widely used to create tissues such as blood vessels, heart, liver, and kidneys. The printing process requires stem cells to form the 3D structure, along with bioinks, such as hydrogels, which act as scaffolds for cell growth. Bioprinting technology includes inkjet printing and laser deposition (Sung et al., 2022; Kwon et al., 2018). Inkjet printer technology is similar to that of a commercial printer, with controlled amounts of liquid being delivered to a predefined location. The printer uses thermal or acoustic forces to place drops of liquid onto a substrate to form the construct. Advantages to this technology include low cost, high availability, high uniformity, high resolution, and high biocompatibility. Despite these advantages, there are limitations that still remain. Printing exposes cells to thermal and mechanical stress, and some cells may not be able to withstand these conditions. Additionally, the biological material would need to be in a liquid state for printing, and then solidify into a 3D structure with proper organization and functionality, which could be unpredictable. Inkjet bioprinting has successfully been used to create functional skin and cartilage (Murphy and Atala, 2014; Skardal et al., 2012; Cui et al., 2012). Laser printing, in contrast, does not exert physical stress on the cells and does not require the biological material to begin in liquid form. As a result, the viability of cells is over 95% after printing, and other cellular functions, such as apoptosis and proliferation, are not affected (Kwon et al., 2018). Laser printing can also be employed for its higher resolution for building microvascular structures, such as lymphatic and blood capillaries (Hwang et al., 2021).

In lymphatic tissue engineering, 3D bioprinting has significantly advanced lymphatic system research. The integration of bioprinted lymphatic vessels has enabled the development of sophisticated tumor models, such as the tumor-on-a-chip system, for studying the tumor microenvironment. For instance, Cao et al. created a tumor-mimetic hydrogel embedded with bioprinted blood and lymphatic vessels. By optimizing vessel permeability through tailored bioink compositions, the researchers tested various combinations of bioprinted blood and lymphatic vessels with different permeabilities to examine drug diffusion through tumor tissue and its subsequent drainage via the lymphatic system. Their findings suggest that this model effectively simulates the tumor microenvironment and complex transport mechanisms, potentially enhancing the accuracy of future cancer drug screening (Cao et al., 2019).

Beyond oncology, bioprinting has also been used to study lymphangiogenesis. Sacrificial bioprinting—a technique where a printed template dissolves or is removed, leaving behind an endothelialized microchannel (Zhang et al., 2018)—has been employed to create a volumetric breast tumor model with embedded multiscale lymphatic vessels. This model facilitated the investigation of tumor-lymphatic interactions, including lymphatic sprouting and breast tumor cell migration. Notably, findings revealed that VEGF-C induced lymphatic endothelial cell proliferation and migration within the system (Liu et al., 2021).

While research on bioprinted lymphatic vessels for lymphedema treatment remains in its early stages, existing studies demonstrate the potential for fabricating functional lymphatic networks and inducing lymphangiogenesis. These advancements suggest that bioprinted lymphatic tissue could 1 day be applied to restore lymphatic drainage and improve outcomes for lymphedema patients.

### 3.6 Extracorporeal shockwave therapy (ESWT)

ESWT has been used for decades across various medical fields to either break down tissue or stimulate tissue repair and regeneration. In urology, it is commonly employed to disintegrate kidney stones, while in orthopedic surgery, it is utilized for treating tendinopathies and non-union fractures (Wang, 2012). More recently, it has emerged as a promising, non-invasive treatment option for lymphedema. It aids in the regeneration of lymphatic valves by increasing cell permeability and facilitating the transport of molecules essential for tissue repair (Alderfer et al., 2018). The therapy delivers pressure waves as mechanical stimuli, exerting physical force on edematous tissue. This physical stress upregulates the expression of VEGF-C and FGF, which are crucial in promoting lymphangiogenesis and enhancing lymphatic drainage (Jia et al., 2021). Numerous preclinical studies have demonstrated the benefits of ESWT. In one study using a rabbit ear lymphedema model, ESWT significantly increased the expression of VEGF-C and VEGFR-3, leading to improved lymphedema outcomes (Kubo et al., 2010). Another study in a rat tail model showed that ESWT induced lymphangiogenesis by upregulating VEGF-C and bFGF, which helped alleviate lymphedema (Serizawa et al., 2011). Additionally, combining ESWT with a VEGF-C hydrogel demonstrated a synergistic effect in a mouse lymphedema model (Kim et al., 2013). Clinically, ESWT has also been applied in patients with breast cancer-related lymphedema (BCRL). Recent studies have shown that ESWT effectively reduces limb volume and enhances patients’ quality of life, with effects lasting up to 6 months post-therapy, making it a long-lasting and potentially cost-effective alternative to conventional lymphedema therapies (Lee et al., 2022; Joos et al., 2021; Cebicci et al., 2016). With its therapeutic benefits and proven effectiveness in both animal and human studies of lymphedema, ESWT holds promise as a valuable addition to lymphatic tissue engineering therapies.

### 3.7 Biomechanical control

Mechanical stimuli and the mechanical environment play a crucial role in lymphatic tissue engineering (Alderfer et al., 2018). Fluid shear stress (FSS), the force exerted by fluid flow on vessel walls, varies depending on the location or type of lymphatic vessel. Initial lymphatics experience lower FSS due to their exposure to interstitial fluid flow, whereas collecting lymphatic vessels encounter greater FSS because of their smooth muscle coverage and pumping capacity. The pumping function of collecting lymphatics generates low levels of nitric oxide (NO), which increases contraction amplitude. Conversely, higher NO concentrations, such as those observed in inflammatory conditions, inhibit both contraction amplitude and frequency. This indicates that higher concentrations of NO, produced by LECs in response to FSS, may contribute to shear stress and dysfunctional lymphatic pumping. This pumping, regulated by smooth muscle coverage surrounding collecting vessels, may play a role in preventing edematous conditions (Angeli and Lim, 2023).

FSS also functions as a key lymphangiogenic mediator, facilitating LEC migration, VEGF-C expression, and lymphatic organization (Swartz and Boardman, 2002). In both lymphatic endothelial cells (LECs) and collecting lymphatic vessels, VEGF-C signals through VEGFR-3 pathways to regulate distinct functions. While its role in lymphangiogenesis has been previously discussed, VEGF-C plays another role in collecting lymphatic vessels. In rat mesenteric collecting lymphatics, VEGF-C treatment has increased contraction frequency, end-diastolic diameter, stroke volume index, and pump flow (Breslin et al., 2007). Moreover, FSS influences LEC shape and alignment. Laminar shear stress (LSS) has been shown to elongate LECs and align fibers in the direction of fluid flow, whereas oscillatory shear stress (OSS) results in cuboidal LECs that are less dependent on fluid flow (Sabine et al., 2012). In addition to FSS, transmural flow significantly impacts the physiological functions of lymphatic vessels. Miteva et al. investigated the effects of transmural flow on lymphatic vessels using both *in vivo* and *in vitro* models. Their findings revealed that transmural flow increases lymphatic permeability to specific macromolecules and promotes dendritic cell transmigration through the vessels. These results suggest that transmural flow may act as an early inflammatory signal for lymphatic vessels, aiding in the delivery of soluble antigens and dendritic cells to lymph nodes (Miteva et al., 2010).

Interstitial flow also plays a part in ECM reorganization, lymphatic cell proliferation, lymphatic capillary morphogenesis, immunity, and peripheral tolerance (Wiig and Swartz, 2012). Lymphatic cells also migrate in the direction of interstitial flow, and do not form into functional capillaries if interstitial flow is severely affected (Rutkowski and Swartz, 2007). Studies have also shown that VEGF-C and interstitial flow work together to promote lymphangiogenesis, with VEGF-C working to induce cell proliferation and migration, while interstitial flow works to promote the organization and functionality of the lymphatic vessels (Goldman et al., 2007). When engineering lymphatic tissue, it is important to take into consideration the scaffolding that encourages interstitial flow, in order to aid in lymphangiogenesis. Helm et al. investigated the effects of slow interstitial flow in hydrogels with varying fibrin and collagen compositions and found that a fibrin-only matrix with slow interstitial flow promotes LEC organization (Helm et al., 2007). This study emphasizes the importance of scaffolding that supports proper interstitial flow in promoting lymphangiogenesis and engineering functional lymphatic tissue.

Mechanotransduction is the process by which cells convert mechanical stimuli into biochemical signals, typically mediated by molecules and transcription factors. Several flow-induced transcription factors have been identified as key mediators of FSS responses in lymphatic vessels. Forkhead box protein C2 (FOXC2) and PROX1 are key regulators of lymphatic vessel and valve development, as well as LEC alignment and organization under LSS and OSS. Studies show that OSS upregulates FOXC2, while the loss of PROX1 disrupts the cuboidal LEC arrangement induced by OSS and instead enhances the elongated LEC arrangement promoted by LSS (Sabine et al., 2012). FOXC2 activates FOXP2, a transcription factor that collaborates with the NFATc1 molecule to regulate collecting vessel morphogenesis and lymphatic valve development (Hernández Vásquez et al., 2021). Conversely, FOXC2-deficient LECs exhibit a disorganized cytoskeleton with disrupted cell-to-cell junctions, leading to abnormal responses to shear stress, increased cell proliferation, and apoptosis (Sabine et al., 2012).

In vitro models, LSS has been shown to enhance calcium levels in LECs via ORAI1, an early mediator of laminar flow. ORAI1 subsequently upregulates KLF2 and KLF4 in flow-activated LECs, leading to the regulation of VEGF-A, VEGF-C, FGFR3, and p57. Mouse embryos deficient in ORAI1 and its downstream molecules KLF2 and KLF4 demonstrated impaired lymphatic development and reduced lymphatic density (Choi et al., 2017). The upstream mechanosensor of ORAI1-mediated calcium entry in LECs is Piezo1. Embryos of Piezo1-knockout mice showed defects in lymphatic sprouting, while adult mice exhibited lymphatic regression. A potential therapeutic option for lymphedema involves Yoda1, a Piezo1 agonist, to stimulate lymphatic regeneration (Choi et al., 2022).

Another critical lymphatic mechanosensor is PECAM-1. Mice deficient in this sensor exhibited impaired flow sensing across LECs, leading to abnormal lymphatic vessel remodeling and valve development. Their lymphatic vessels displayed irregular, enlarged branches, while LECs appeared randomly oriented with abnormal valves. *In vivo*, Pecam1 and Sdc4 knockout mice developed significant edema, highlighting the necessity of both molecules for proper lymphatic vessel and valve formation (Wang et al., 2016).

Disruption in these mechanical forces contributes to the pathophysiology of lymphedema. Impaired lymphatic drainage alters the biomechanical environment, leading to endothelial dysfunction and progressive tissue changes, exacerbating lymphedema symptoms (Angeli and Lim, 2023). While conventional lymphedema diagnostic measures, such as circumferential limb measurement or water displacement, provide limited information on underlying tissue changes, recent studies suggest that biomechanical tools can offer a more precise approach for detecting lymphedema at earlier stages. For instance, the MyotonPro device, a noninvasive instrument used to measure skin stiffness, elasticity, oscillation frequency, relaxation time, and creep (the ratio of deformation to relaxation time) can assess biomechanical changes in lymphedematous tissue. Glassman et al. used this device to compare skin biochemical parameters between affected and unaffected arms in patients with breast cancer-related lymphedema. They found that on average, the affected arms exhibited decreased skin stiffness and oscillation frequency, and increased relaxation time and creep compared to the contralateral unaffected arms (Glassman et al., 2022). Similarly, Naczk et al. used MyotonPro to analyze the viscoelastic property of tissue in breast cancer-related lymphedema patients compared to controls, with similar findings to the previous study (Naczk et al., 2022). These findings suggest the potential of biomechanical assessment tools in improving early disease detection, monitoring of disease progression, and guiding personalized treatment plans for patients with lymphedema.

Beyond diagnostics, biomechanical control also holds promise for therapeutic strategies in lymphedema management. One key application is the optimization of compression therapy. By incorporating biomechanical measurements of tissue stiffness and elasticity, compression therapy can be personalized to ensure optimal pressure distribution for intermittent pneumatic compression devices (Zaleska et al., 2018). Additionally, tissue engineering offers a promising approach by leveraging the connection between the extracellular matrix (ECM) and the mechanical environment, both of which regulate lymphatic structure and function (Angeli and Lim, 2023). By developing biomechanical scaffolds that mimic the ECM, tissue engineering can enhance the biomechanical environment of the lymphatic system, ultimately promoting lymphatic vessel regeneration and stabilization.

## 4 Staged lymphatic tissue engineering

Staged lymphatic tissue engineering, using host sites or external bioreactors, marks significant progress in immunotherapy and regenerative medicine by enhancing adaptive immune responses. Creating lymphatic tissue-like organoids can provide potential application into immune system reconstruction and personalized immunotherapy, specifically for immunodeficient patients. In immunocompromised (SCID) mouse models, introducing antigen-specific lymphocytes can initiate adaptive immunity. This approach involves embedding engineered stromal cells within biocompatible scaffolds to create artificial lymphoid tissues, which are then transplanted to host sites, like the renal subcapsular space in mice. When stromal cells expressing lymphotoxin alpha (LTa) and activated dendritic cells (DCs) are embedded within a collagen-based scaffold, they attract lymphocytes and form organized lymphoid structures, such as distinct B- and T-cell clusters and structures resembling high endothelial venules (HEV) (Suematsu and Watanabe, 2004). These antigen-specific immune responses can therefore serve as a potential application for immune regulation in lymphedema patients as well.

A crucial component in this engineering method is the development of scaffolds that mimic natural lymphoid microenvironments. Collagen-based scaffolds have shown effectiveness by providing a supportive matrix for cell migration, interaction, and proliferation. The addition of activated DCs further promotes lymphocyte accumulation and organization, establishing conditions similar to natural lymphoid tissues. This organization supports immune cell clustering and promotes the formation of germinal centers and functional dendritic cell networks, facilitating antigen presentation and B-cell maturation (Suematsu and Watanabe, 2004). This adaptability highlights the therapeutic potential for treating immune deficiencies or chronic infections, where engineered lymphoid structures could enable localized, controlled immune responses.

External bioreactors provide an alternative staging approach that is valuable for pre-conditioning engineered tissues before implantation. Bioreactors enable controlled cell growth and differentiation under specific conditions, allowing for the generation of mature lymphoid structures that are ready for integration into the host. One of the key advantages of bioreactors is their ability to replicate the dynamic environment of lymphoid organs, which is essential for creating functional tissues (Selden and Fuller, 2018). In lymphatic tissue engineering, bioreactors function as external vessels that simulate *in vivo* conditions necessary for cellular development. They offer structured scaffolding for stromal cells, DCs, and other immune cells, supporting interactions that lead to organized structures resembling secondary lymphoid organs (Giese et al., 2006). When combined with artificial antigen-presenting cells, bioreactors can generate cytotoxic T-cells from naïve T-cells, making them a promising tool for tumor immunotherapy, artificial lymphoid tissue engineering, and transplantation to promote lymphatic regeneration (Kobayashi and Watanabe, 2010). Combining these strategies with pro-lymphangiogenic factors, such as VEGF-C, surgical interventions like vascularized lymph node transfer (VLNT) or lymphovenous anastomosis, and optimized scaffold materials could provide a comprehensive and multifaceted therapeutic approach to lymphedema.

## 5 Challenges

Artificial tissue engineering has rapidly advanced, but key challenges remain. A major hurdle is selecting the right scaffold, essential for structural support, cell proliferation, and tissue regeneration (Kanapathy et al., 2014; Alderfer et al., 2018). The ideal material must mimic natural anatomy and physiology to generate functional tissue. Another challenge is obtaining enough cells for scaffold seeding, particularly in older or severely ill patients. Harvesting LECs can be invasive and yield limited quantities, requiring time-consuming cell expansion. Induced pluripotent stem cells (iPSCs) or adult progenitor cells offer alternatives, but protocols ensuring proper functionality need further research (Sung et al., 2022; Alderfer et al., 2018; Ikada, 2006). Controlling growth factor delivery is also critical, as improper targeting or release can cause complications. Binding growth factors to a carrier can enhance precision, but selecting the right carrier is essential for effectiveness (Jia et al., 2021; Ikada, 2006). Lymphatic tissue engineering presents specific challenges due to the complex structure of the lymphatic system, which consists of capillaries, vessels, and lymph nodes. Replicating this intricate architecture is critical to its function and poses significant challenges. Unlike blood vessel engineering, lymphatic vessel engineering must account for unidirectional flow, lymphatic valves, and the diverse structures throughout the system (Alderfer et al., 2018). One of the most difficult aspects is creating functional lymphatic valves—bileaflet valves that respond to lymphatic fluid and prevent retrograde flow (Kanapathy et al., 2014). Research is currently exploring the use of 3D printing combined with bioreactors to stimulate valve development and maturation (Jia et al., 2021).

Lymphatic grafts have primarily focused on recreating microvascular structures, such as capillaries. However, scaling up these approaches to engineer larger vessels and lymph nodes is crucial for treating conditions like lymphedema and immunologic diseases. Regenerating larger lymphatic vessels is crucial for efficiently returning substantial amounts of lymphatic fluid to circulation (Jia et al., 2021), while regenerating lymph nodes presents a promising avenue for personalized immunotherapy and long-term treatment options for immunodeficient patients (Suematsu and Watanabe, 2004). Engineering larger vessels is essential to overcoming the limitations of current lymphedema therapies, such as lymphovenous anastomosis, which relies on the presence of functional lymphatic vessels for success (Drobot et al., 2024). Similarly, engineering lymph nodes could address the shortcomings of vascularized lymph node transfer, which carries the risk of iatrogenic lymphedema at the donor site (Schaverien et al., 2018). By providing an unlimited, autologous alternative, engineered lymph nodes could eliminate the need for donor-site tissue, reducing complications and expanding treatment accessibility.

Larger tissues require more complex scaffold designs, precise control over cell organization and differentiation, and efficient oxygen and nutrient delivery (Sung et al., 2022). Additionally, ensuring endothelialization of lymphatic vessels is essential for regulating fluid flow and immune cell transport. While lymphatic grafts do not carry a risk of thrombosis like vascular grafts, low flow rates and scaffolding materials can increase the risk of coagulation (Sung et al., 2022; Kanapathy et al., 2014; Dai et al., 2010). To enhance endothelialization in blood vessel grafts, ligands such as arginine-glycine-aspartic acid (RGD) have been studied for their ability to improve cell adhesion (Hernández Vásquez et al., 2021). A similar approach could be applied to LECs to identify ligands that enhance lymphatic graft endothelialization (Kanapathy et al., 2014). Techniques like surface topography and nanostructuralization, which increase surface area, have also been explored in blood vascular grafts. Adapting these methods for lymphatic vessels could help create more functional lymphatic grafts (Sung et al., 2022; Miller et al., 2004).

Another concern in lymphatic tissue engineering is the potential link between lymphangiogenesis and immune rejection (Alderfer et al., 2018). Lymphangiogenesis has been speculated to contribute to autoimmune-related chronic inflammatory disorders and transplant rejection (von der Weid et al., 2011; Kerjaschki et al., 2004). It is therefore crucial to understand the relationship between lymphangiogenesis and the immune system to mitigate risks associated with engineered lymphatic tissue.

Despite these challenges, there is optimism about the future of artificial lymphatic tissue engineering. Advances in lymphatic development, cell biology, and biomaterial science, along with innovations in 3D printing and other engineering techniques, are paving the way for effective treatments for lymphedema and other debilitating lymphatic disorders.

## 6 Advancements and the future of artificial tissue engineering

In artificial lymphatic tissue engineering, significant advancements have helped tackle challenges like lymphedema. Nanofibrillar collagen scaffolds, which mimic the extracellular matrix of lymphatic tissues, are biocompatible and biodegradable, serving as a supportive structure for lymphatic regeneration. These scaffolds, seeded with lymphatic endothelial cells (LECs) and growth factors, have shown promising results in treating secondary lymphedema (Yesantharao and Nguyen, 2022). Decellularized lymph nodes, created by removing cells while retaining structural and biochemical cues, offer a biomimetic approach to tissue-engineered lymph nodes (Sung et al., 2022). Stem cell-based therapies, particularly those using adipose-derived stem cells (ADSCs), have shown potential in differentiating into LECs and secreting lymphangiogenic factors (Forte et al., 2021). 3D bioprinting, specifically for lymphatic tissue, is advancing with bioinks designed to support lymphatic cell growth, although achieving the fine resolution needed for capillary-sized vessels remains a challenge (Mirshafiei et al., 2024; Moysidou et al., 2021). Furthermore, organs-on-chips technology, such as lymph nodes-on-chips, is emerging as a platform to model lymphatic function and disease *in vitro* (Shanti et al., 2021; Wang et al., 2024).

Looking ahead, the future of artificial tissue and lymphatic tissue engineering is promising, with a focus on overcoming current limitations and creating more complex tissue structures, such as whole organs and functional lymphatic vessels. Future research will likely center on developing biomaterials that replicate the intricate mechanical and biological properties of these structures (Sung et al., 2022; Liu et al., 2024). Improved bioinks for 3D bioprinting will be crucial in achieving the precision necessary for regenerating complex tissues (Mirshafiei et al., 2024). Gene editing technologies, such as CRISPR/Cas9, offer exciting potential for modifying cells and scaffolds to enhance tissue regeneration by creating genetically modified cells that promote new tissue growth (De Chiara et al., 2024). Nanotechnology may play a pivotal role in developing targeted drug delivery systems aimed at lymphatic tissues, allowing for more precise treatments of lymphatic disorders (He et al., 2023). Personalized medicine, driven by patient-specific genetic information, will enable the creation of highly compatible tissues, reducing the risk of rejection (De Chiara et al., 2024). *In situ* bioprinting may allow for less invasive treatments with greater precision through direct tissue printing within the body (Mirshafiei et al., 2024). Finally, integrating engineered tissues with immune and other body systems will be critical to ensuring their long-term functionality, particularly in lymphatic tissue engineering, where these systems are closely interconnected (Suematsu and Watanabe, 2004). These advancements will enable more personalized, effective treatments for a wide range of conditions, offering significant improvements in patient outcomes.

## 7 Discussion

The integration of biomaterials, lymphangiogenic factors, and regenerative medicine approaches has led to significant advancements in the field of artificial lymphatic tissue engineering. These innovations hold the potential to transform the treatment of lymphedema and other lymphatic disorders.

The use of biomaterials such as hydrogels, decellularized extracellular matrices, and biodegradable and non-biodegradable polymers has provided a solid foundation for lymphatic tissue engineering. These materials offer biocompatibility, structural support, and the potential for controlled degradation, which are crucial for developing functional lymphatic networks (Sung et al., 2022; Drobot et al., 2024). The successful use of lymphangiogenic factors, such as VEGF-C and fibroblast growth factors, has also facilitated the development of lymphatic vessels and improved lymphangiogenesis in preclinical models (Jia et al., 2021; Shin et al., 2006). Furthermore, the use of stem-cell-based therapies, particularly adipose-derived stem cells (ADSCs), has shown promise in differentiating into lymphatic endothelial cells and promoting lymphatic repair (Forte et al., 2021). Additionally, advances in 3D bioprinting and microfluidic technologies have enabled the precise fabrication of artificial lymphatic networks, allowing researchers to replicate capillary-like structures with increasing accuracy. These developments provide an opportunity to create patient-specific grafts tailored to individual anatomical and physiological needs, ultimately improving the feasibility of clinical applications (Cao et al., 2019; Zhang et al., 2018; Liu et al., 2021).

Despite these strengths, significant limitations remain. One of the primary challenges is achieving the structural complexity of the lymphatic system, which consists of intricate capillary networks, collecting vessels, and lymph nodes. Current engineering approaches often struggle to replicate the dynamic interactions between these structures, particularly in the formation of functional, unidirectional valves and the prevention of fluid stasis. Scalability also remains a significant obstacle, as many tissue-engineered lymphatic constructs have only been tested in small animal models. Translating these findings into human applications requires the development of larger, more complex scaffolds capable of supporting long-term lymphatic function. Additionally, ensuring the sustained viability and integration of engineered tissues within host environments remains a critical concern. Regulatory approval poses another barrier to clinical implementation. Engineered tissues must meet stringent safety and efficacy requirements before they can be used in patients. The variability in scaffold materials, cell sources, and fabrication techniques complicates the standardization process, making it challenging to develop universally accepted guidelines for lymphatic tissue engineering (Jia et al., 2021; Kanapathy et al., 2014; Ikada, 2006).

Several knowledge gaps persist in the field of lymphatic tissue engineering. While preclinical studies have demonstrated the potential for scaffold-based lymphangiogenesis, long-term studies assessing the functional stability of these engineered tissues are lacking. The durability of implanted lymphatic constructs, their ability to integrate with existing lymphatic networks, and their effectiveness in reducing chronic inflammation and fibrosis in lymphedema patients require further investigation (Alderfer et al., 2018). Additionally, there is limited research on the impact of patient-specific factors, such as genetic predisposition, immune responses, and comorbidities, on the success of engineered lymphatic tissues. Personalized medicine approaches that consider these factors could improve patient outcomes, but more research is needed to develop individualized treatment strategies (De Chiara et al., 2024).

Lymphatic scaffolds have shown significant promise in promoting lymphangiogenesis and restoring lymphatic function in preclinical models. Among these, nanofibrillar collagen scaffolds (NCSs) have demonstrated effectiveness in reducing limb volume and enhancing lymphatic regeneration, while decellularized lymph nodes, when repopulated with stem cells, offer a potential avenue for restoring immune function in patients with compromised lymphatic systems. Additionally, hydrogel-based delivery systems have successfully facilitated localized growth factor release, supporting sustained lymphangiogenesis and improving therapeutic outcomes.

To fully realize the clinical potential of these approaches, ongoing research must focus on optimizing scaffold composition and mechanical properties to enhance therapeutic efficacy and improve clinical translation. Despite significant advancements in biomaterials, lymphangiogenic factors, and scaffold-based engineering, several challenges remain, including scalability, regulatory approval, and long-term viability. Overcoming these hurdles will require interdisciplinary collaboration, rigorous preclinical studies, and patient-specific strategies to refine engineered lymphatic tissues for widespread clinical use. As the field continues to evolve, these innovations have the potential to revolutionize the treatment of lymphatic disorders, ultimately improving patient outcomes and quality of life.
